# Susceptibility to heat wave-related mortality: a follow-up study of a cohort of elderly in Rome

**DOI:** 10.1186/1476-069X-8-50

**Published:** 2009-11-12

**Authors:** Patrizia Schifano, Giovanna Cappai, Manuela De Sario, Paola Michelozzi, Claudia Marino, Anna Maria Bargagli, Carlo A Perucci

**Affiliations:** 1 Department of Epidemiology, Local Health Authority Rome E, Rome, Italy

## Abstract

**Background:**

Few studies have identified specific factors that increase mortality during heat waves. This study investigated socio-demographic characteristics and pre-existing medical conditions as effect modifiers of the risk of dying during heat waves in a cohort of elderly residents in Rome.

**Methods:**

A cohort of 651,195 residents aged 65 yrs or older was followed from 2005 to 2007. During summer, heat wave days were defined according to month-specific thresholds of maximum apparent temperature. The adjusted relative risk of dying during heat waves was estimated using a Poisson regression model including all the considered covariates. Risk differences were also calculated. All analyses were run separately for the 65-74 and 75+ age groups.

**Results:**

In the 65-74 age group the risk of dying during heat waves was higher among unmarried subjects and those with a previous hospitalization for chronic pulmonary disease or psychiatric disorders. In the 75+ age group, women, and unmarried subjects were more susceptible to heat. Furthermore, a higher susceptibility to heat among those with previous hospitalization for diabetes, diseases of the central nervous system (CNS), psychiatric disorders and cerebrovascular diseases resulted from risk differences.

**Discussion:**

Results showed a higher susceptibility to heat among those older than seventy-five years, females and unmarried. Pre-existing health conditions play a different role among the two considered age groups. Moreover, compared with previous studies the pattern of susceptibility factors have slightly changed over time. For the purposes of public health programmes, susceptibility should be considered as time, space and population specific.

## Introduction

High temperatures and heat waves have been associated with short term increases in mortality worldwide. In Europe, the exceptionally long and severe heat wave of summer 2003 found local communities substantially unprepared to cope with such an extreme event. Epidemiological research and public health efforts have been focused to better understand the health impact of heat and of the individual factors that confer susceptibility to heat stress. Several countries throughout Europe have put into place a series of preventive measures, but only few are specifically targeted to high-risk individuals [1].

During heat waves the greatest burden in terms of mortality and morbidity is in specific population subgroups more susceptible to the effect of heat; they are represented by people with impaired physiological and behavioural responses to heat due to their old age [2-5], to the presence of chronic illnesses [3-7], to limited social contacts [6], to living alone [5,8], to low socio-economic conditions [3,5], and limited access to air conditioning [6]. While for some of these factors, such as old age, there is strong evidence and physiological plausibility for their role in increasing the vulnerability to heat waves, for others, like specific health conditions and factors as social, behavioural and environmental conditions, evidence is still not conclusive and somewhat controversial, and more research is needed.

Under a public health point of view a clear understanding of these factors is important to ensure the effectiveness of prevention programs.

Until now, most of the evidence regarding factors that modify the health effects of heat waves derives from case-control studies [6,9], and, more recently, from case-only and case-crossover studies [5,10-13]. However, in such studies the design itself doesn't allow to estimate comparable relative risks of dying during heat waves for different subgroups of interest [14].

Under a public health point of view a clear understanding of factors affecting heat wave related mortality is important to properly target prevention programs. Moreover after the 2003 heat wave in Europe the introduction of prevention programs may have modified the determinants of the population's susceptibility to high temperature [15,16].

In Italy the National Department of Civil Protection and the Ministry of Health implemented a national heat prevention program [17], and national guidelines for the prevention of heat health effects have been defined, targeted to high risk subgroups http://www.ccm-network.it. In Lazio, the region surrounding Roma, the heat response plan is centered on active surveillance of high risk elders (65+ years), identified using available health information systems [18].

As a part of the Lazio prevention program for summer 2008, we carried out a longitudinal analysis of heat wave mortality between 2005 and 2007 with the primary aim of identifying susceptibility factors. The inclusion of the most recent years was to account for the possible changes in the spectrum of susceptibility factors, as well as changes in the impact of heat waves on mortality due to the effect of the heat prevention plan. We describe the results of these analyses, and discuss possible public health implications.

## Materials and methods

### Study Population

We analyzed a cohort of residents in Rome aged 65 yrs or older as of the 1^st ^of May 2005; this cohort was followed up for the subsequent two years, until the 15^th ^of September 2007. Each year new residents aged 65 years and older were added to the cohort, and subjects who died or changed residence by the 1^st ^of May were removed. Information on resident's age, gender, and marital status was extracted from the Population Registry of the Roma Municipal Office. A small area-based (census tract) socio-economic status indicator using 2001 census data was also used [19].

### Health Status

We selected a list of 13 groups of diagnoses, known to be associated with an increased mortality risk from high temperatures (Table 1) [3,5,7,12,20]. Information about the prevalence of past hospitalizations for each of these conditions in the study population was extracted by linking the regional hospital discharge files (Regional Hospital Information System) (HIS) [21], and the Municipality Population Registry, using individual social security numbers as the linkage key. We considered hospitalizations or day-hospital visits for each diagnostic group, reported either as primary causes of admission or as contributing diagnoses. Subjects were classified using 13 dichotomous variables, each one indicating if they sought medical treatment for one of the 13 diagnoses or not.

**Table 1:** Person years and number of deaths by population characteristics and age groups.

	All 65+ ages		65-74 ages		75+ ages	
	***Person yrs***	***Deaths***	***Person yrs***	***Deaths***	***Person yrs***	***Deaths***

	***(n = 641147)***	**18609**	***(n = 348015)***	**4139**	***(n = 293132)***	**14470**

	***%***		***%***		***%***	

Age						

*65-74*	*54.3*	4139				

*75-84*	*34.4*	7599				

*85-94*	*9.8*	5781				

*95+*	*1.6*	1090				

Gender						

*Female*	*59.1*	10112	*55.6*	1700	*63.3*	8412

*Male*	*40.9*	8497	*44.4*	2439	*36.7*	6058

Marital Status						

*Married*	*56.2*	8090	*68.5*	2825	*41.5*	5265

*Not married, widowed, divorced*	*43.8*	10519	*31.5*	1314	*58.5*	9205

SES						

*Medium/High*	*81.9*	15126	*80.5*	3144	*83.6*	11982

*Low*	*17.1*	3323	*18.6*	954	*15.4*	2369

*Missing*	*1.0*	160	*0.9*	41	*1.0*	119

HDD (Hospitalization Diagnosed Disease)						

*(ICD9-CM)*						

*Malignant neoplasm*	*3.9*	4510	*3.7*	1840	*4.1*	2670

*(140-208)*						

*Diabetes mellitus*	*3.9*	2237	*3.4*	576	*4.5*	1661

*(250)*						

*Other CNS disorders*	*1.9*	1530	*1.2*	335	*2.7*	1195

*(330-349)*						

*Ischaemic heart diseases*	*4.1*	2445	*3.3*	521	*5.1*	1924

*(410-414)*						

*Conduction disorders*	*1.1*	580	*0.6*	105	*1.5*	475

*(426)*						

*Cardiac dysrhythmias*	*3.5*	2557	*2.3*	437	*4.9*	2120

*(427)*						

*Heart failure*	*1.5*	1846	*0.8*	301	*2.4*	1545

*(428)*						

*Other cardiovascular diseases*	*11.5*	5476	*9.3*	1221	*14.2*	4255

*(390-429 exept 410-414;426;427;428)*						

*Chronic pulmonary diseases*	*3.1*	2344	*2.2*	495	*4.2*	1849

*(490-496)*						

*Acute and chronic liver diseases*	*0.7*	593	*0.7*	229	*0.7*	364

*(570-572)*						

*Renal failure*	*1.3*	1689	*0.7*	319	*1.9*	1370

*(584-588)*						

*Psychiatric disorders*	*1.2*	837	*0.8*	150	*1.6*	687

*(290-299;300.4;301.1;309.0;309.1;311)*						

*Cerebrovascular diseases*	*3.9*	2591	*2.4*	445	*5.6*	2146

*(430-438)*						

Number of HDD for any other causes						

*0-1*	*97.6*	17774	*97.6*	3864	*97.6*	13910

*2-3*	*2.2*	715	*2.2*	213	*2.3*	502

*4+*	*0.2*	120	*0.2*	62	*0.2*	58

For each subject, the number of Hospitalization Diagnosed Disease (HDD) over the previous two years for any cause, other than those specified on our list, was also considered as an indicator of general health status.

### Exposure

The exposure of interest was a heat wave episode, defined using maximum apparent temperature (Tappmax), an index of discomfort based on air and dew point temperatures [22]. Tappmax was defined as the maximum daily value of apparent temperatures.

A heat wave episode was defined when Tappmax rises above the monthly threshold, defined by the Heat Health Watch Warning System (HHWWS) [17], (May: 28.5, June: 32.5°C; July: 33.5°C; August/September: 34.5°C). for two or more consecutive days. Daily weather data from Ciampino airport station were obtained from the Italian Air Force Meteorological Service, during summer (1^st ^of May until the 15^th ^of September).

### Outcome

Vital status was determined each year, for each subject through a record linkage with the Regional Registry of Causes of Death (Regional Mortality Register) [23] using individual social security numbers. We considered all deaths from non-injury causes (International Classification of Diseases, 9th Revision [ICD-9]: 1-799).

The outcome of interest was every death occurred during a heat episode or in the following three days; the three extra days were considered to account for the lag effect of heat on mortality.

### Statistical Analysis

We assumed that age modifies the set of factors that interact with the heat-mortality relationship. Hence we analyzed the 65-74 and the over 74 year old separately.

We used a Poisson regression model to estimate the adjusted relative risk of mortality during heat wave days versus not heat wave days. We run a model including an indicator variable for heat wave days, age, gender, socio-economic status, civil status, presence or absence of each of the selected groups of diagnosis and the number of hospitalizations for conditions not included in the previous list.

Through a manual backward stepwise procedure we then selected a model including only variables significantly associated with mortality. Age, gender and presence/absence of heat wave were forced into the model. Interaction terms between each of the included covariates and heat-wave were added afterwards.

We calculated the relative risks (RR) of dying on heat wave and non heat wave days for each of the modalities of the covariates and the REM (Relative Effect Modification) index. REM index was the ratio of the relative risks of dying during a heat wave for each of the covariates considered, comparing each category to the reference one [5]. A significant REM index (p-value lesser than 0.20) means that the specific covariate acts as an effect modifier of the heat-mortality relationship. Risk differences (RD) were computed as the difference between the model estimates of absolute risks of mortality in the presence and absence of heat wave for each modality of the considered covariates. A comparison between RDs relative to different modalities of the same variable was performed to evaluate a possible effect modification on an additive scale. The analysis was performed using SAS [24].

## Results

We enrolled a total of 651,195 subjects over the three-year period, 574,192 of which were included in the first year. A total of 26,634 subjects (5%) were excluded from the analysis due to incomplete civil status information. The excluded subjects were comparable to the study population, except for a higher percentage of subjects over 95 years of age; no deaths were observed in this subgroup. The analyzed cohort represented 203,626 person-years of heat wave exposure (including the three post heat wave lag days) and 437,520 person-years of non heat wave exposure. Table 1 reports the population characteristics. About 50% of the analyzed population was older than 75 and about 40% were male.

There were more females and a higher percentage of unmarried people in the over 75-year-old group than in the younger group. The 75+ group also had a higher prevalence of previous hospitalizations for all the considered diseases compared to the 65-74 age group. We observed 4,139 deaths in the 65-74 age group and 14,470 deaths among the over 74 year olds.

Table 2 reports the average Tappmax observed in the study period.

**Table 2:** Temperature distribution and number of days by month and heat wave (years 2005-2007).

	May		Jun		Jul		Aug	
	**Heat wave**		**Heat wave**		**Heat wave**		**Heat wave**	

	**no**	**yes**	**no**	**yes**	**no**	**yes**	**no**	**yes**

min	14.3	28.8	17.3	31.2	24.4	32.6	20.8	32.9

max	27.3	33.5	32.4	38.4	34.2	39.1	34.3	39.7

20 pct	18.7	29.2	20.6	33.4	27.8	34.4	26.4	34.3

50 pct	21.1	29.9	26.7	34.6	29.9	35.3	28.8	35.5

90 pct	25.7	30.9	31.0	38.1	33.1	38.5	32.3	39.1

n(dd)	82	11	68	22	51	42	80	13

On average there was an 8°C difference between the median temperature on heat wave and non heat wave days in May and June, about a 5°C difference in July, and 6°C in August. There were no heat waves, and therefore no results reported for the month of September in the years we studied. According to the exposure definition single days with Tappmax over the monthly threshold were included in the "non heat waves days". This contributed to a slight overlap of temperatures observed between heat wave and non heat wave days.

### The heat wave effect

We observed a crude relative risk of dying during heat waves compared to non heat waves of 1.08 (IC95%: 1.02-1.16) in the 65-74 age group, and of 1.15 (IC95%: 1.11-1.18) in the over 74 age group.

### 65-74 age group

Results suggested that the excess in mortality during heat waves was higher among those who were previously hospitalized for a chronic pulmonary disease, and to a smaller extent, for psychiatric disorders and among those not married, widowed or divorced (Tables 3 and 4). For these same factors we observed also an increased excess in the absolute numbers of deaths during heat wave days compared to non heat wave days. Subjects previously hospitalized for malignant neoplasm, cardiac arhythmias and renal failure had lower relative risks of dying during heat wave days. Although not statistically significant, having been previously hospitalized for diabetes, ischemic diseases or liver diseases showed a similar protective effect; while socio-economic status does not modify the relationship under study.

**Table 3:** Mortality rate (MR), risk difference (RD), adjusted mortality relative risk (RR) and relative effect modification (REM) index for socio-demographic covariates modalities by heat wave. 65-74 and 75+ age groups.

	65-74 ages										75+ ages									
	***Deaths***		MR *1000		RD *1000	***(95% IC)***	***RR***	***(95% IC)***	***REM Index***	***p-value***	***Deaths***		MR *1000		RD *1000	***(95% IC)***	***RR***	***(95% IC)***	***REM Index***	***p-value***

	***Heat wave***		***Heat wave***								***Heat wave***		***Heat wave***							

	***no***	***yes***	***no***	***yes***							***no***	***yes***	***no***	***yes***						

																				

*Total*	2753	1386	11.6	12.6	1.0	*(0.2; 1.8)*	1.08	*(1.02 - 1.16)*			9427	5043	47.2	54.1	6.9	*(5.1; 8.7)*	1.15	*(1.11 - 1.18)*		

																				

Age																				

*75-84*											5004	2595	33.2	37.0	3.8	*(2.1; 5.5)*	1.11	*(1.06 - 1.17)*	*1.00*	

*85-94*											3733	2048	87.8	102.4	14.7	*(9.4; 19.9)*	1.17	*(1.11 - 1.23)*	*1.05*	*0.196*

*95+*											690	400	101.7	124.9	23.3	*(8.9; 37.7)*	1.23	*(1.08 - 1.39)*	*1.10*	*0.149*

																				

Gender																				

*Female*	1124	576	8.5	9.4	0.9	*(0.0; 1.8)*	1.11	*(1.00 - 1.22)*	*1.00*		5406	3006	42.7	50.9	8.2	*(6.1; 10.3)*	1.19	*(1.14 - 1.24)*	*1.00*	

*Male*	1629	810	15.4	16.5	1.1	*(-0.3; 2.5)*	1.07	*(0.99 - 1.17)*	*0.96*	*0.666*	4021	2037	54.8	59.5	4.6	*(1.5; 7.7)*	1.08	*(1.03 - 1.14)*	*0.91*	*0.008*

																				

Marital Status																				

*Married*	1891	934	11.6	12.3	0.7	*(-0.3; 1.6)*	1.06	*(0.98 - 1.15)*	*1.00*		3494	1771	42.2	45.5	3.3	*(0.7; 5.8)*	1.07	*(1.02 - 1.14)*	*1.00*	

*Not married, widowed, divorced*	862	452	11.5	13.1	1.6	*(0.2; 3.0)*	1.14	*(1.02 - 1.28)*	*1.08*	*0.323*	5933	3272	50.7	60.2	9.5	*(7.1; 12.0)*	1.18	*(1.13 - 1.24)*	*1.10*	*0.008*

																				

SES																				

*Medium/High*	2088	1056	10.9	11.9	1.0	*(0.1; 1.8)*	1.09	*(1.01 - 1.18)*	*1.00*		7807	4175	46.7	53.5	6.8	*(4.9; 8.8)*	1.14	*(1.10 - 1.19)*	*1.00*	

*Low*	634	320	14.4	15.6	1.3	*(-0.8; 3.3)*	1.09	*(0.95 - 1.25)*	*1.00*	*0.999*	1531	838	49.7	58.1	8.4	*(3.7; 13.1)*	1.17	*(1.07 - 1.27)*	*1.03*	*0.668*

*Missing*	31	10	13.7	9.5	-4.2	*(-11.8; 3.4)*	0.69	*(0.34 - 1.40)*	*0.64*	*0.202*	89	30	46.6	33.6	-13	*(-28.4; 2.5)*	0.72	*(0.48 - 1.10)*	*0.63*	*0.031*

**Table 4:** Results on the 65-74 age group.

	65-74 ages									
	***Deaths***		MR *1000		RD *1000	***(95% IC)***	***RR***	***(95% IC)***	***REM Index***	***p-value***

	***Heat wave***		***Heat wave***							

	***no***	***yes***	***no***	***yes***						

HDD										

*Malignant neoplasm*										

*no*	1503	796	6.6	7.5	0.9	*(0.3; 1.5)*	1.14	*(1.05 - 1.24)*	*1.00*	

*yes*	1250	590	141.9	143.7	1.8	*(-12.2; 15.8)*	1.02	*(0.93 - 1.13)*	*0.89*	*0.092*

*Diabetes mellitus*										

*no*	2363	1200	10.3	11.3	1.0	*(0.2; 1.7)*	1.10	*(1.02 - 1.17)*	1.00	

*yes*	390	186	47.7	48.9	1.2	*(-7.3; 9.6)*	1.03	*(0.87 - 1.23)*	0.94	*0.550*

*Other CNS disorders*										

*no*	2534	1270	10.8	11.7	0.9	*(0.1; 1.6)*	1.08	*(1.01 - 1.16)*	1.00	

*yes*	219	116	75.2	87.0	11.7	*(-7.0; 30.4)*	1.15	*(0.92 - 1.44)*	1.07	*0.615*

*Ischaemic heart diseases*										

*no*	2396	1222	10.4	11.5	1.0	*(0.3; 1.8)*	1.10	*(1.03 - 1.18)*	1.00	

*yes*	357	164	45.0	44.6	-0.4	*(-8.7; 7.9)*	1.01	*(0.84 - 1.21)*	0.92	*0.398*

*Conduction disorders*										

*no*	2683	1351	11.4	12.3	1.0	*(0.2; 1.7)*	1.08	*(1.02 - 1.16)*	1.00	

*yes*	70	35	45.7	49.7	4.0	*(-15.7; 23.6)*	1.09	*(0.73 - 1.61)*	1.00	*0.992*

*Cardiac dysrhythmias*										

*no*	2443	1259	10.5	11.7	1.2	*(0.4; 1.9)*	1.11	*(1.04 - 1.19)*	1.00	

*yes*	310	127	57.2	50.5	-6.7	*(-17.6; 4.1)*	0.90	*(0.73 - 1.10)*	0.81	*0.055*

*Heart failure*										

*no*	2552	1286	10.8	11.7	0.9	*(0.2; 1.7)*	1.09	*(1.02 - 1.16)*	1.00	

*yes*	201	100	106.9	115.0	8.2	*(-18.8; 35.1)*	1.10	*(0.87 - 1.40)*	1.01	*0.892*

*Other cardiovascular diseases*										

*no*	1935	983	9.0	9.8	0.8	*(0.1; 1.6)*	1.09	*(1.01 - 1.18)*	1.00	

*yes*	818	403	37.0	39.4	2.4	*(-2.2; 7.0)*	1.06	*(0.95 - 1.2)*	0.97	*0.697*

*Chronic pulmonary diseases*										

*no*	2435	1209	10.5	11.2	0.7	*(0.0; 1.5)*	1.07	*(1.00 - 1.15)*	1.00	

*yes*	318	177	59.9	72.8	12.9	*(0.3; 25.5)*	1.23	*(1.03 - 1.48)*	1.15	*0.155*

*Acute and chronic liver diseases*										

*no*	2595	1315	11.0	12.0	1.0	*(0.2; 1.8)*	1.09	*(1.02 - 1.17)*	1.00	

*yes*	158	71	95.7	93.1	-2.7	*(-28.9; 23.6)*	1.00	*(0.76 - 1.33)*	0.92	*0.567*

*Renal failure*										

*no*	2529	1291	10.7	11.8	1.1	*(0.3; 1.8)*	1.10	*(1.03 - 1.18)*	1.00	

*yes*	224	95	127.7	115.7	-12.0	*(-40.6; 16.7)*	0.93	*(0.73 - 1.18)*	0.85	*0.177*

*Psychiatric disorders*										

*no*	2658	1331	11.3	12.2	0.9	*(0.1; 1.7)*	1.08	*(1.01 - 1.15)*	1.00	

*yes*	95	55	47.2	59.2	12.0	*(-6.3; 30.2)*	1.27	*(0.91 - 1.77)*	1.18	*0.350*

*Cerebrovascular diseases*										

*no*	2455	1239	10.6	11.5	0.9	*(0.2; 1.7)*	1.09	*(1.02 - 1.17)*	1.00	

*yes*	298	147	51.8	54.9	3.1	*(-7.5; 13.8)*	1.08	*(0.88 - 1.31)*	0.99	*0.907*

Number of HDD for any other causes										

*0-1*	2555	1309	11.0	12.1	1.1	*(0.4; 1.9)*	1.11	*(1.04 - 1.18)*	1.00	

*2-3*	151	62	29.4	26.2	-3.2	*(-11.2; 4.8)*	0.89	*(0.66 - 1.20)*	0.80	*0.161*

*4+*	47	15	98.4	68.9	-29.5	*(-74.3; 15.3)*	0.68	*(0.38 - 1.21)*	0.61	*0.099*

Mortality rate (MR), risk difference (RD), adjusted mortality relative risk (RR) and relative effect modification (REM) index for hospitalization diagnosed diseases (HDD) modalities by heat wave.

### 75+ age group

Relative risks showed that the excess in mortality during heat waves is significantly higher in females, and for all those not married, widowed or divorced (Table 3). Rate ratios showed that the effect of heat on mortality increases with age and is greater for those who had four or more hospitalizations for other causes in the previous two years, although they were not statistically significant. However, having had four or more hospitalizations was associated to a higher absolute number of deaths, as shown by RDs (Table 5).

**Table 5:** Results on the 75+ age group.

	75+ ages									
	***Deaths***		MR *1000		RD *1000	***(95% IC)***	***RR***	***(95% IC)***	***REM Index***	***p-value***

	***Heat wave***		***Heat wave***							

	***no***	***yes***	***no***	***yes***						

HDD										

*Malignant neoplasm*										

*no*	7627	4173	39.8	46.7	6.9	*(5.2; 8.5)*	1.17	*(1.13 - 1.21)*	1.00	

*yes*	1800	870	217.4	224.3	6.9	*(-11.0; 24.9)*	1.03	*(0.95 - 1.12)*	0.88	*0.006*

*Diabetes mellitus*										

*no*	8340	4469	43.7	50.1	6.5	*(4.7; 8.2)*	1.15	*(1.10 - 1.19)*	1.00	

*yes*	1087	574	122.2	137.8	15.6	*(2.2; 29.0)*	1.12	*(1.02 - 1.24)*	0.98	*0.734*

*Other CNS disorders*										

*no*	8657	4618	44.5	50.9	6.4	*(4.6; 8.1)*	1.14	*(1.10 - 1.18)*	1.00	

*yes*	770	425	144.8	171.4	26.6	*(7.4; 45.9)*	1.18	*(1.04 - 1.32)*	1.04	*0.625*

*Ischaemic heart diseases*										

*no*	8126	4420	42.8	49.9	7.1	*(5.3; 8.8)*	1.16	*(1.12 - 1.20)*	1.00	

*yes*	1301	623	127.6	131.3	3.7	*(-8.7; 16.1)*	1.03	*(0.94 - 1.13)*	0.89	*0.020*

*Conduction disorders*										

*no*	9107	4888	46.3	53.2	6.9	*(5.2; 8.7)*	1.15	*(1.11 - 1.19)*	1.00	

*yes*	320	155	104.0	108.6	4.6	*(-15.9; 25.2)*	1.04	*(0.86 - 1.26)*	0.91	*0.337*

*Cardiac dysrhythmias*										

*no*	7999	4351	42.1	49.0	7.0	*(5.2; 8.7)*	1.16	*(1.12 - 1.21)*	1.00	

*yes*	1428	692	147.3	152.7	5.4	*(-8.3; 19.2)*	1.03	*(0.94 - 1.13)*	0.89	*0.019*

*Heart failure*										

*no*	8385	4540	43.0	49.9	6.9	*(5.2; 8.6)*	1.16	*(1.12 - 1.20)*	1.00	

*yes*	1042	503	215.7	221.7	6.1	*(-17.3; 29.5)*	1.03	*(0.92 - 1.14)*	0.89	*0.035*

*Other cardiovascular diseases*										

*no*	6643	3572	38.8	44.6	5.9	*(4.2; 7.6)*	1.15	*(1.11 - 1.20)*	1.00	

*yes*	2784	1471	97.9	110.9	13.0	*(6.2; 19.7)*	1.13	*(1.07 - 1.20)*	0.98	*0.643*

*Chronic pulmonary diseases*										

*no*	8186	4435	42.8	49.6	6.9	*(5.1; 8.6)*	1.16	*(1.11 - 1.20)*	1.00	

*yes*	1241	608	146.1	154.7	8.6	*(-6.1; 23.4)*	1.06	*(0.96 - 1.17)*	0.91	*0.095*

*Acute and chronic liver diseases*										

*no*	9172	4934	46.2	53.3	7.0	*(5.3; 8.8)*	1.15	*(1.11 - 1.19)*	1.00	

*yes*	255	109	188.4	172.5	-15.8	*(-55.6; 24.0)*	0.91	*(0.73 - 1.14)*	0.79	*0.041*

*Renal failure*										

*no*	8503	4597	43.4	50.2	6.9	*(5.2; 8.6)*	1.16	*(1.12 - 1.20)*	1.00	

*yes*	924	446	243.3	249.6	6.3	*(-21.7; 34.3)*	1.03	*(0.92 - 1.15)*	0.89	*0.048*

*Psychiatric disorders*										

*no*	8980	4803	45.7	52.3	6.7	*(4.9; 8.4)*	1.14	*(1.10 - 1.18)*	1.00	

*yes*	447	240	136.5	158.2	21.7	*(-2.0; 45.4)*	1.16	*(0.99 - 1.35)*	1.02	*0.894*

*Cerebrovascular diseases*										

*no*	8033	4291	42.6	48.7	6.2	*(4.4; 7.9)*	1.14	*(1.10 - 1.19)*	1.00	

*yes*	1394	752	125.5	144.3	18.8	*(6.6; 31.1)*	1.15	*(1.05 - 1.26)*	1.00	*0.896*

Number of HDD for any other causes										

*0-1*	9061	4849	46.5	53.3	6.8	*(5.0; 8.6)*	1.14	*(1.10 - 1.18)*	1.00	

*2-3*	331	171	72.4	81.5	9.1	*(-5.4; 23.6)*	1.11	*(0.92 - 1.34)*	0.97	*0.772*

*4+*	35	23	93.2	134.5	41.3	*(-21.7; 104.4)*	1.44	*(0.85 - 2.44)*	1.27	*0.388*

Mortality rate (MR), risk difference (RD), adjusted mortality relative risk (RR) and relative effect modification (REM) index for hospitalization diagnosed diseases (HDD) modalities by heatwave. 75+ age groups.

We observed more deaths attributable to heat waves among those who had a previous hospitalization for diabetes, diseases of the central nervous system, psychiatric disorders and cerebrovascular diseases.

Conversely, there was a protective effect observed among subjects with a previous hospitalization for a wide range of conditions, including malignant neoplasm, ischemic diseases, hearth rhythm disorders, heart failure, chronic pulmonary diseases, liver diseases and renal failure. As in the 65-74 age group, socio-economic status did not modify the effect.

## Discussion

This study analysed factors associated to heat related mortality in a cohort of elderly subjects in the summers of 2005-2007 in Roma. In these years prevention programs for the effects of heat on health were active in the city. The analysis was performed separately for the 65-74 and the 75+ age group. Overall, the impact of heat wave on mortality was higher in the older age group; moreover, females and unmarried subjects resulted to be more susceptible to heat waves effects.

Results from the 65-74 age group identified only previous hospitalizations for chronic pulmonary disease as a significant effect modifier; however, subjects with at least one hospitalization for psychiatric disorders showed a higher excess in mortality during heat wave days. In the 75+ age group, a higher susceptibility to heat was observed among those with previous hospitalization for diabetes, diseases of the central nervous system, psychiatric disorders and cerebrovascular diseases, but only from the risk differences analysis. Our results confirmed that age acts as an effect modifier of the associations under study, and suggested that the underlying mechanisms that determine degrees of susceptibility to heat waves might vary with age.

As far as we know, this is one of the few studies to analyze a prospective cohort with individual socio-demographic and health information on the elderly, stratified by age.

Compared to the more widely used case crossover design, our study has the advantage of allowing us to estimate comparable relative risk of dying during heat waves for different covariates of interest. Estimates were obtained from the interaction term of a model including the heat wave effect, the covariate effect and the interaction term, and adjusted for a pool of covariates. Furthermore, this study design allowed us not only to compute the relative risk such as in a cross-over design but also the absolute risk. As a consequence we could evaluate both the multiplicative effect modification of the factors considered as well as the impact in terms of absolute number of excess of deaths during a heat wave. This has enabled us to identify a series of health conditions that modify the heat-mortality relationship on an additive scale but not on a multiplicative scale.

The higher relative risk of mortality during heat waves in subjects with previous hospitalizations for a chronic respiratory illness is supported by previous case-only, case-crossover and time-series studies [10,12,25,26]. In patients with COPD an exposure to extreme heat might induce hyperventilation leading to dyspnea and to mechanical and cardiovascular effects [27]; furthermore, some heat-induced physiological changes such as dehydration might represent a risk factor of broncho-pulmonary disorders [28]. Such mechanism offers an explanation of the higher rates of hospitalization for respiratory causes observed by a European multicity study [29]. The higher risk of dying among people with psychiatric disorders is supported by previous studies [3,12], and may be explained by the inability of these people to care for themselves [3], and by the side effects of some neuroleptic drugs [30].

In this as in other studies [4,5,12], the effect of heat waves results stronger among those older than 75 years. This can be attributed to concurrent factors such as pre-existing chronic diseases, physical fitness level, and attenuated physiological responses to heat such as lower sweat gland outputs, decreased skin blood flow and reduced cardiac outputs [31].

Results from both age groups showed that those who were hospitalized in the previous two years had a smaller risk of dying during heat waves than non previously hospitalized subjects. For some specific health conditions, such as cancer or other severe chronic illnesses, this lower mortality risk could be due to the fact that these patients received greater medical attention. On the other hand, it has been suggested that the lower risk of dying during heat waves for pre-hospitalized people should be interpreted more as an additive than a multiplicative effect, since the absolute risk of dying among those who had an hospitalization during the previous two years is presumably higher than in the not hospitalized group [6].

As reported by previous studies [5], we did not observe any effect modification by socio-economic conditions in either of the two considered age groups. It should be noted that our indicator of socio-economic status is derived from a census tract level study [19], therefore misclassification of individual exposure may have affected our findings toward the null.

The comparison of our results with previous studies on risk factors for heat related mortality in the 65+ age group in Rome before 2003 [5,12] shows a slight change in the pattern of the concurrent clinical conditions associated to a higher susceptibility to heat. Heart failure and heart conduction disorders were not currently identified as risk modifiers as they had been previously [5,12], whereas diabetes and diseases of the CNS were identified in this study as having a higher number of deaths during heat waves. These changes may have resulted from the implementation of prevention programs targeted to high risk population in Rome in recent years [17,18].

Some limitations of this study need to be discussed. Even if we have considered a three-year period cohort of all the residents in Rome, the high number of covariates included in the model determine a problem of power in the analysis. Among the 65-74 years old in particular we might not have had enough power to detect all the effect modifiers.

We used cause-specific hospital admissions as markers of specific diseases. This has some intrinsic limitations since not all conditions considered result in a hospital admission and in this case the use of additional sources of health data (i.e. outpatient care, pharmaceutical care) could have provided a more accurate measure of the prevalence of the disease in our population. Furthermore, it should be noted that the use of hospital admissions could provide a biased picture of morbidity since it reflects not only morbidity levels but also provider-specific factors and the availability of primary care and out-patient services [32].

In this study, we used "population-averaged" temperatures collected at a single monitor (the nearest airport), but no potential measurement error should have affected our effect estimates since it was possibly of the same entity in heat wave and non-heat wave days and cancelled by comparisons. Possible sources of exposure bias are however the micro-urban variations in heat effect that are influenced by the underlying socio-economic conditions and the summer migratory patterns that finds the elderly and ill people and those of low socio-economic status more often in the city. This bias is difficult to quantify and to be adjusted for from the available data.

## Conclusion

Our study is one of the few to analyze the potential modification effects of a wide range of health conditions and of socio-demographic factors in a large cohort of elderly subjects in the years following the introduction of prevention programs aimed to prevent the health effects of heat waves. Results suggest that prevention programs should mainly target people over 74 years of age, women and unmarried people. Specifically, in the 65-74 years old age group people with chronic pulmonary diseases should be particularly monitored during heat waves, while further analysis should be done to better understand the role of previous hospitalizations for cerebrovascular, CNS, psychiatric disorders and diabetes among those older than 75 years. In conclusion, for the purposes of public health programmes susceptibility should be considered time, space and population specific, and the identification of prognostic factors should be performed repeatedly over time.

## Competing interests

The authors declare that they have no competing interests.

## Authors' contributions

PS contributed to defining the objectives of the analysis, designed and supervised the analysis the analysis and drafted the paper; GC carried out the analysis and participated in the preparation of the paper; MDS contributed to writing and revised the paper; PM contributed to defining the objectives of the analysis and to writing and revising the paper; CM contributed to data management and revised the paper; AMB participated to the methodological discussion and revised the paper; CAP participated to the discussion of methodology and results and revised the paper. All authors read and approved the final version.

## References

[B1] Kovats RS Ebi KL Heatwaves and public health in Europe Eur J Public Health 2006 16 592 599 10.1093/eurpub/ckl049 16644927

[B2] Johnson H Kovats RS McGregor G Stedman J Gibbs M Walton H The impact of the 2003 heat wave on daily mortality in England and Wales and the use of rapid weekly mortality estimates Euro Surveill 2005 10 168 171 16088043

[B3] Michelozzi P de'Donato F Bisanti L Russo A Cadum E DeMaria M D'Ovidio M Costa G Perucci CA The impact of the summer 2003 heat waves on mortality in four Italian cities Euro Surveill 2005 10 161 165 16088045

[B4] Fouillet A Rey G Laurent F Pavillon G Bellec S Ghihenneuc-Jouyaux C Clavel J Jougla E Hémon Denis Excess mortality related to the August 2003 heat wave in France Int Arch Occup Environ Health 2006 80 16 24 16523319 10.1007/s00420-006-0089-4 PMC1950160

[B5] Stafoggia M Forastiere F Agostini D Biggeri A Bisanti L Cadum E Caranci N de'Donato F De Lisio S De Maria M Michelozzi P Miglio R Pandolfi P Picciotto S Rognoni M Russo A Scarnato C Perucci CA Vulnerability to heat-related mortality: a multicity, population-based, case-crossover analysis Epidemiology 2006 17 315 323 10.1097/01.ede.0000208477.36665.34 16570026

[B6] Vandentorren S Bretin P Zeghnoun A Mandereau-Bruno L Croisier A Cochet C Ribe' ron J Siberan I Declercq B Ledrans M August 2003 heat wave in France: risk factors for death of elderly people living at home Eur J Publ Health 2006 16 583 591 10.1093/eurpub/ckl063 17028103

[B7] Conti S Masocco M Meli P Minelli G Palummeri E Solimini R Toccaceli V Vichi M General and specific mortality among the elderly during the 2003 heat wave in Genova (Italy) Environ Res 2007 103 267 274 10.1016/j.envres.2006.06.003 16890219

[B8] Canoui-Poitrine F Cadot E Spira A Groupe regional Canicule: Excess deaths during the August 2003 heat wave in Paris, France Rev Epidemiol Sante Publique 2005 54 127 135 10.1016/S0398-7620(06)76706-2 16830967

[B9] Naughton MP Henderson A Mirabelli MC Kaiser R Wilhelm JL Kieszak SM Rubin CH McGeehin MA Heat-related mortality during a 1999 heat wave in Chicago Am J Prev Med 2002 22 221 227 10.1016/S0749-3797(02)00421-X 11988377

[B10] Schwartz J Who is sensitive to extremes of temperature? A case-only analysis Epidemiology 2005 16 67 72 10.1097/01.ede.0000147114.25957.71 15613947

[B11] Medina-Ramon M Schwartz J Temperature, temperature extremes, and mortality: a study of acclimatisation and effect modification in 50 US cities Occup Environ Med 2007 64 827 833 10.1136/oem.2007.033175 PMC2095353 17600037

[B12] Stafoggia M Forastiere F Agostini D Caranci N de'Donato F Demaria M Michelozzi P Miglio R Rognoni M Russo A Perucci CA Factors affecting in-hospital heat-related mortality: A multi-city case-crossover analysis J Epidemiol Community Health 2008 62 209 215 10.1136/jech.2007.060715 18272735

[B13] Bell ML O'Neill MS Ranjit N Borja-Aburto VH Cifuentes LA Gouveia NC Bell ML Vulnerability to heat-related mortality in Latin America: a case-crossover study in Sao Paulo, Brazil, Santiago, Chile and Mexico City, Mexico Int J Epidemiol 2008 37 796 804 18511489 10.1093/ije/dyn094 PMC2734062

[B14] Skrondal A Interaction as departure from additivity in case-control studies: a cautionary note Am J Epidemiol 2003 158 251 258 10.1093/aje/kwg113 12882947

[B15] Tan J Zheng Y Song G Kalkstein LS Kalkstein AJ Tang X Heat wave impacts on mortality in Shangai, 1998 and 2003 Int J Biometeorol 2007 51 193 200 10.1007/s00484-006-0058-3 17039379

[B16] Fouillet A Rey G Wagner V Laaidi K Empereur-Bissonnet P Tertre AL Frayssinet P Bessemoulin P Laurent F Crouy-Chanel PD Jougla E Hémon D Has the impact of heat waves on mortality changed in France since the European heat wave of summer 2003? A study of the 2006 heat wave Int J Epidemiol 2008 37 309 317 18194962 10.1093/ije/dym253 PMC2652641

[B17] de'Donato FK Michelozzi P Bargagli AM Di Gennaro M D'Ippoliti D Leonardi M Marino C Schifano P Perucci CA The Italian heat/health warning system for prevention of heat health effects: Evaluation of Summer 2008 [Abstracts: ISEE 20th Annual Conference, Pasadena, California, October 12-16, 2008: Contributed Abstracts] Epidemiology 2008 19 Supplement S287 S288

[B18] Bargagli A Michelozzi P De Sario M Chierchini P Casali V Cervelli S Barcaioli E Tanfetti S Perucci CA Summer 2007 heat waves in Rome: Results of the prevention programme based on general practitioner surveillance [Abstracts: ISEE 20th Annual Conference, Pasadena, California, October 12-16, 2008: Contributed Abstracts] Epidemiology 2008 19 Supplement S245

[B19] Cesaroni G Agabiti N Rosati R Forastiere F Perucci CA An index of socioeconomic position based on 2001 Census, Rome [Article in Italian] Epidemiol Prev 2006 30 352 357 17333691

[B20] Semenza JC McCullough JE Flanders WD McGeehin MA Lumpkin JR Excess hospital admissions during the July 1995 heat wave in Chicago Am J Prev Med 1999 16 269 277 10.1016/S0749-3797(99)00025-2 10493281

[B21] Regione Lazio. DGR 2 dicembre n. 9158 Riorganizzazione del sistema informativo ospedaliero (SIO). Bollettino Ufficiale della Regione Lazio n. 6 parte 1, 28 febbraio 1994

[B22] Kalkstein LS Valimont KM An evaluation of summer discomfort in the United States using a relative climatological index Bull Am Meteorolog Soc 1986 67 842 848 10.1175/1520-0477(1986)067<0842:AEOSDI>2.0.CO;2

[B23] Regione Lazio Direttive e Modalità di Attuazione di un Nuovo Sistema Informativo di Mortalità. Istituzione presso le USL di un Registro Nominativo delle Cause di Morte

[B24] SAS Statistical Software for Linux, release 8.02.02 1999 SAS Institute Inc., Cary, NC, USA

[B25] Diaz J Jordán A Garcia R López C Alberdi JC Hernández E Otero A Heat waves in Madrid 1986-1997: effects on the health of the elderly Int Arch Occup Environ Health 2002 75 163 170 10.1007/s00420-001-0290-4 11954983

[B26] Ishigami A Hajat S Kovats RS Bisanti L Rognoni M Russo A Paldy A An ecological time-series study of heat related mortality in three European cities Environ Health 2008 7 5 18226218 10.1186/1476-069X-7-5 PMC2266730

[B27] Sprung CL Heat Stroke: Modern approach to an ancient disease Chest 1980 77 461 462 10.1378/chest.77.4.461 7357963

[B28] Kalhoff H Mild dehydration: a risk factor of broncho-pulmonary disorders? Eur J Clin Nutr 2003 57 Suppl 2 S81 87 10.1038/sj.ejcn.1601906 14681718

[B29] Michelozzi P Accetta G De Sario M D'Ippoliti D Marino C Baccini M Biggeri A Anderson HR Katsouyanni K Ballester F Bisanti L Cadum E Forsberg B Forastiere F Goodman PG Hojs A Kirchmayer U Medina S Paldy A Schindler C Sunyer J Perucci CA on behalf of the PHEWE collaborative group High temperature and hospitalizations for cardiovascular and respiratory causes in 12 European cities Am J Respir Crit Care Med 2009 179 383 389 10.1164/rccm.200802-217OC 19060232

[B30] Kilbourne EM Choi K Jones S Thacker SB The Field Investigation Team Risk factors for heatstroke - A case-control study JAMA 1982 47 3332 3336 10.1001/jama.247.24.3332 7087076

[B31] Kenney WL Munce TA Invited review: Aging and human temperature regulation J Appl Physiol 2003 95 2598 2603 14600165 10.1152/japplphysiol.00202.2003

[B32] Hansell A Bottle A Shurlock L Aylin P Accessing and using hospital activity data J Public Health Med 2001 23 51 56 10.1093/pubmed/23.1.51 11315695

